# A Solution to Ambiguities in Position Estimation for Solenoid Actuators by Exploiting Eddy Current Variations

**DOI:** 10.3390/s20123441

**Published:** 2020-06-18

**Authors:** Niklas König, Matthias Nienhaus

**Affiliations:** Laboratory of Actuation Technology, Saarland University, 66123 Saarbrücken, Germany; nienhaus@lat.uni-saarland.de

**Keywords:** solenoid, position estimation, self-sensing, sensorless, eddy currents

## Abstract

Position estimation techniques for solenoid actuators are successfully used in a wide field of applications requiring monitoring functionality without the need for additional sensors. Most techniques, which also include standstill condition, are based on the identification of the differential inductance, a parameter that exhibits high sensitivity towards position variations. The differential inductance of some actuators shows a non-monotonic dependency over the position. This leads to ambiguities in position estimation. Nevertheless, a unique position estimation in standstill condition without prior knowledge of the actuator state is highly desired. In this work, the eddy current losses inside the actuator are identified in terms of a parallel resistor and are exploited in order to solve the ambiguities in position estimation. Compared to other state-of-the-art techniques, the differential inductance and the parallel resistance are estimated online by approaches requiring low implementation and computation effort. Furthermore, a data fusion algorithm for position estimation based on a neural network is proposed. Experimental results involving a use case scenario of an end-position detection for a switching solenoid actuator prove the uniqueness, the precision and the high signal-to-noise ratio of the obtained position estimate. The proposed approach therefore allows the unique estimation of the actuator position including standstill condition suitable for low-cost applications demanding low implementation effort.

## 1. Introduction

Solenoid actuators have proven to be a simple and robust actuation principle for various applications such as valves and electromechanical switches. Being based on the reluctance principle, they offer linear motion with large strokes and high forces. Due to their simplicity, such actuators are produced in high quantities for low prices. With the recent tendencies towards condition monitoring and predictive maintenance, mainly under the concept of Industry 4.0, also monitoring of the state and the condition of such actuators gains interest. In particular, a knowledge of the actuator position is required in order to detect if the solenoid actuates correctly under varying external loads. Usually, position information is obtained by additional position sensors such as linear variable differential transformers (LVDTs) or encoders, which increase the size and the cost of the actuation system significantly. The usage of additional sensors can be avoided by applying position estimation techniques for actuator monitoring and control [1,2,3,4,5], thus allowing an implementation on low-cost applications. Moreover, even sophisticated actuation systems containing position sensors benefit from such approaches since a redundancy can be achieved with the merit of increasing the functional safety [6].

State-of-the-art techniques for sensorless position detection of solenoid actuators can be divided into three categories: observer based on the back-induced electromotive force (back-EMF) [7,8], identification of the differential inductance [4,9,10,11,12,13,14,15,16,17,18,19,20,21,22,23,24,25,26] and identification of eddy current losses [23,27,28]. Observer-based techniques exploit the back-EMF induced inside an actuator during movement [6,7,8], thus delivering precise and unique estimates of the position when the actuator is moving at middle or high speed. At standstill or low speed condition, such approaches are not able to operate due to a vanishing back-EMF.

The differential inductance of a solenoid actuator shows a significant dependence on the actuator position and is exploited in various techniques that allow the estimation even at standstill condition. It can only be identified when the actuator is persistently excited, which can be achieved by approaches injecting an additional excitation signal into the actuator [4,6,9,10]. Nonetheless, the injected signal causes significant acoustic noise and force ripples. Therefore, more sophisticated works use the inherent current ripple caused by a pulse width modulated (PWM) switching electronics. In such cases, current ripples are always present during driving and no further injection signal is required. The current ripple can be measured and processed by different approaches. Techniques calculating the current derivative numerically [11,12,13,14,15,16,17] obtain an estimate with reduced measurement and calculation effort, but with low signal-to-noise ratio (SNR) due to the usage of a numerical derivative on a noisy current signal. In order to improve the SNR of the position estimate, oversampling approaches [18,19,20,21,22] acquire a large amount of current samples and perform online regression methods such as a least-mean squares (LMS) algorithm. Despite the benefit of increasing the SNR significantly, such techniques require additional sampling and computational effort, thus making their implementation on low-cost actuators difficult. Other approaches [23,24,25,26] are based on the analog processing of the current ripple with the aim of increasing the SNR and decreasing the computational effort, thus allowing the implementation on cost-critical actual inductance towards position variation at the complete speed range. Therefore, a majority of works prefers the evaluation of this parameter. Nevertheless, on solenoid actuators that operate in strong magnetic saturation, the differential inductance shows a non-monotonic characteristic over the position ([29] p. 21), thus resulting in ambiguous position estimates. The work [23] avoids these ambiguities by using look-up tables together with a prior knowledge of the actuator position and moving direction. Despite its simplicity, such an approach needs always an initialization procedure and can lose tracking capability at high speeds.

The state-of-the-art works [23,27,28] model eddy current losses inside an actuator by a lumped parameter system. In general, all the above-mentioned techniques, disregarding the estimation approach, exhibit the strong sensitivity of the different parameter model containing a leakage resistor in parallel to the main inductance of the solenoid. This lumped parameter also exhibits a position dependence. In particular, the work [23] observes that the characteristic of that resistor over the position range is monotonic and therefore allows a unique position estimate. However, due to a high measurement variance, the work avoids the exploitation of that parameter. Instead, the works [27,28] identify the parallel resistance by means of a model adaptive reference system (MRAS) and validate the usability of this parameter for position estimation in experimental tests. In order to improve the estimation performance, the information is merged with an estimate of the differential inductance by means of binary decision-making. Further improvements are obtained by increasing the sample time of current and voltage measurements [27,28]. Nevertheless, such high sampling and computational efforts seem not to be adequate in low-cost applications.

As mentioned above, the preferred parameter used for position estimation is the differential inductance, which has the demerit of an ambiguous position estimate. Such ambiguity can be resolved by the identification of a lumped parallel resistor that represents eddy current losses. While some works based on the differential inductance are especially optimized for low computational and sampling effort, similar approaches for the parallel resistance do not exist. Therefore, the synthesis of an estimator for the parallel resistor with low demands on computational power and sampling rate is desired, especially for solenoid actuators that are placed in the low-cost segment. Moreover, the parallel resistance suffers from a large variance in identification, making data fusion with another parameter like the differential inductance necessary for a successful implementation of a position estimator. State-of-the-art works apply a binary decision-making algorithm for this, hence a more sophisticated approach with an internal weighting of the parameter values seems to promise more accurate results with higher SNR.

In this work, a position estimator based on the identification of the differential inductance and the parallel resistor is proposed with the focus lying on a resource-efficient identification of those parameters. Before introducing the methods used for identification, a detailed electromagnetic model of a solenoid actuator is derived, and the current ripple induced in such an actuator is analyzed by taking into account the presence of eddy currents. The response of the current ripple during a PWM switching voltage transient is exploited for deriving a resistance estimator. For a resource-efficient identification of the differential inductance, the Integrator-Based Direct Inductance Measurement (IDIM) technique, known from prior works [25,26], is presented and summarized. Both parameter information are merged by means of a multilayer perceptron neural network, allowing a unique position estimation by weighting the different information. Finally, an experimental use case scenario involving an end-position detection is presented and experimentally validated in terms of uniqueness, accuracy and SNR.

## 2. Analysis of Current Ripples Inside Solenoid Actuators Considering Eddy Currents

In the following, current ripples inside solenoid actuators are analyzed under the consideration of eddy currents. A magnetic circuit model including eddy currents is proposed and its interaction with the electrical model of a solenoid is examined. Finally, an investigation of the response of the electromagnetic model to a PWM voltage is made with the particular focus lying on the switching time instants, where the effect of the eddy currents is significant.

### 2.1. Magnetic Circuit Model Including Eddy Currents

The magnetic flux path of actuators can be modeled by means of magnetic reluctances ([29] p. 15):

(1) $$R_{m}=\frac{l_{eff}}{\mu_{0}\mu_{r}A_{eff}},$$

with $l_{eff}$ and $A_{eff}$ being the effective length and cross-section of a piece of material with a certain relative magnetic permeability $\mu_{r}$. In particular, for an actuator containing an air gap with the length *x*, the air gap reluctance is position-dependent and can be described as ([29] p. 69):

(2) $$R_{mx}\left(x\right)=\frac{x}{\mu_{0}A_{eff}}.$$

Since the relative magnetic permeability $\mu_{r}$ is derived from the B-H-curve of the magnetic material, it depends on the actual magnetic field and therefore on the working point on the B-H-curve.

Solenoid actuators are usually made of non-laminated soft magnetic materials with a certain electric conductivity. Therefore, eddy currents are induced under varying magnetic flux. The presence of such eddy currents can be modeled by the so-called magnetic inductances ([29] p. 155). The term inductance is appropriate since the eddy currents delay the rise of the magnetic flux inside a magnetic material in response to an applied magneto-motive force (MMF) similar like an electric inductance delays the rise of the current due to an applied voltage. A magnetic inductance can be calculated as:

(3) $$L_{m}=\frac{\sigma A_{ed}}{l_{ed}},$$

where σ indicates the electrical conductivity of the material and $A_{ed}$ as well as $l_{ed}$ denote the effective area and length of the piece of material in which the eddy currents are induced. The term of the magnetic inductance represents the electrical conductance of that piece of material.

The plunger of solenoid actuators represents a voluminous piece of soft magnetic material, where the creation of eddy currents is delayed between the outer parts and the inner parts of the plunger. In particular, this phenomena resembles the skin effect, where the eddy currents in the inner parts are delayed since they not only are affected by the original magnetic field, but also from the field that is induced by the eddy currents in the outer parts ([30] p. 51). Therefore, the plunger can be represented, accordingly to [29], as a cascade of low pass filters made of different reluctances and magnetic inductances.

**Figure 1:**
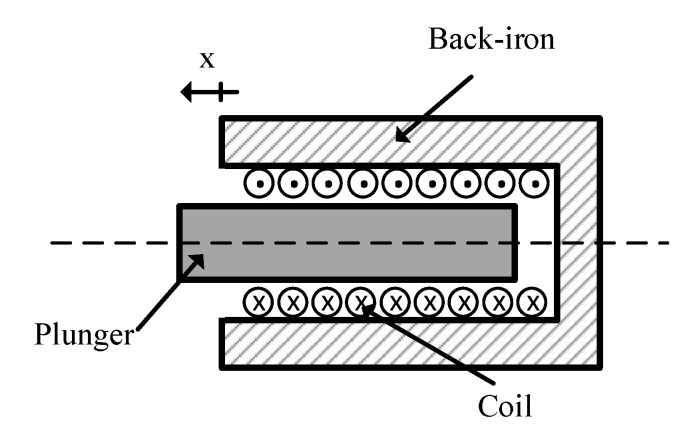
Schematic figure of a solenoid actuator, consisting of coil, plunger and back-iron.

**Figure 2:**
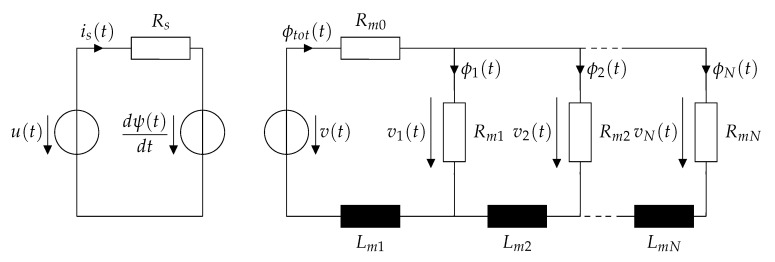
Full electromagnetic equivalent circuit of a solenoid actuator including cascades of reluctances and magnetic inductances representing the eddy currents.

Figure 1 illustrates the basic structure of a solenoid actuator, consisting of coil, plunger and back-iron. The magnetic flux flows through all those elements. Therefore, the equivalent circuit of a solenoid actuator, including the electrical and magnetic subsystem, can be considered as shown in Figure 2. In this model, magnetic leakage fluxes are not considered.

The equivalent circuit is divided into an electrical subsystem, consisting of an electrical voltage *u* and current $i_{s}$, a series resistance $R_{s}$ as well as a back-induced voltage. The magnetic circuit consists of a magnetic voltage source *v* as well as a total flux $\phi_{tot}$ and the position-dependent reluctance $R_{m0}\left(x\right)$, representing the air gap reluctance and the back-iron reluctance. The plunger instead is modeled by a cascade of a number of *N* magnetic reluctances and inductances [29], which all depend on the plunger position *x*. For a better comprehension and modeling, the following calculations will take place in the Laplace domain with the Laplace value denoted as *s*. By applying the Kirchhoff rules, the total flux $\Phi_{tot}\left(s\right)$ inside the actuator

(4) $$\Phi_{tot}\left(s\right)=\sum_{j=1}^{N}\Phi_{j}\left(s\right),$$

and the magnetic voltage *V* can be obtained:

(5) $$V\left(s\right)=R_{m0}\Phi_{tot}\left(s\right)+sL_{m1}\Phi_{tot}\left(s\right)+R_{m1}\Phi_{1}\left(s\right).$$

For each single RL element inside the cascade, it can be calculated:

(6) $$V_{i}\left(s\right)=\Phi_{i}\left(s\right)R_{mi}=V_{i-1}\left(s\right)-sL_{mi}\sum_{j=i}^{N}\Phi_{j}\left(s\right),$$

where $V_{i}\left(s\right)$ is the voltage over the i-th RL element and $\Phi_{i}\left(s\right)$ denotes the flux through the reluctance $R_{mi}$. The flux $\Phi_{i}\left(s\right)$ inside the i-th branch can therefore be expressed as:

(7) $$\Phi_{i}\left(s\right)=\frac{V_{i-1}\left(s\right)-sL_{mi}\sum_{j=i}^{N}\Phi_{j}\left(s\right)}{R_{mi}}.$$

### 2.2. Electrical Circuit Model

The electrical and the magnetic circuit are coupled through the electromotive force (EMF) $\frac{d\psi (t)}{dt}$ on one side and the MMF $v\left(t\right)=\int_{l_{1}}^{l_{2}}H\left(t\right)dl$ on the other side. By considering the flux linkage as

(8) $$\Psi_{tot}\left(s\right)=W\cdot \Phi_{tot}\left(s\right),$$

and the relation between the current and the MMF

(9) $$V\left(s\right)=\int_{l_{1}}^{l_{2}}H\left(s\right)dl=\sum_{W}I_{s}\left(s\right)=W\cdot I_{s}\left(s\right),$$

the number of windings *W* of the coil can be considered in the calculations. Equation (7) allows the calculation of all the fluxes inside a solenoid actuator with a certain number of *N* cascaded RL elements. Nevertheless, it is visible that the calculation of the flux inside one branch requires the knowledge of the fluxes in the other branches, which increases calculation and modeling effort significantly. For sake of simplicity, only the cases for N=1 and N=2 will be evaluated here and a generalization for higher orders will be discussed.

For the case of one RL element (N=1), the magnetic circuit can be mathematically described as:

(10) $$V\left(s\right)=R_{m0}\Phi_{tot}\left(s\right)+R_{m1}\Phi_{tot}\left(s\right)+sL_{m1}\Phi_{tot}\left(s\right).$$

Applying Equations (8) and (9) yields to

(11) $$WI_{s}\left(s\right)=\frac{1}{W}\left(R_{m0}+R_{m1}+sL_{m1}\right)\Psi_{tot}\left(s\right).$$

By means of algebraic manipulation, the transfer function of the magnetic circuit can be obtained:

(12) $$\frac{\Psi_{tot}\left(s\right)}{I_{s}\left(s\right)}=\frac{W^{2}}{R_{m0}+R_{m1}+sL_{m1}}=\frac{\Gamma (s)}{\Upsilon (s)}\;\mathrm{with}\;\deg\left(\Gamma \left(s\right)\right)=0\;\mathrm{and}\;\deg\left(\Upsilon \left(s\right)\right)=1.$$

Thus, the transfer function of the magnetic circuit with one eddy current element has no zeros and one pole. The electrical system can be modeled by applying the Kirchhoff rules as:

(13) $$U\left(s\right)=R_{s}I_{s}\left(s\right)+s\Psi_{tot}\left(s\right)=R_{s}I_{s}\left(s\right)+s\frac{\Gamma (s)}{\Upsilon (s)}I_{s}\left(s\right).$$

By defining the electrical transfer function:

(14) $$\frac{I(s)}{U(s)}=\frac{\Upsilon (s)}{R_{s}\Upsilon \left(s\right)+s\Gamma \left(s\right)}=\frac{\Xi (s)}{\Lambda (s)}\;\mathrm{with}\;\deg\left(\Xi \left(s\right)\right)=1\;\mathrm{and}\;\deg\left(\Lambda \left(s\right)\right)=1,$$

It is visible that the resulting transfer function of the electrical circuit has one pole and one zero. For sake of generalization, the polynomial of the nominator is denoted as Ξ(s) and the one of the denominator as Λ(s). In the electrical transfer function, one zero is added in relation to the transfer function of the magnetic circuit.

By using Equation (7), the transfer function of the magnetic circuit for a cascade of two eddy current elements (N=2) can be derived:

(15) $$\frac{\Psi_{tot}\left(s\right)}{I_{s}\left(s\right)}=\frac{W^{2}\left(R_{m1}+R_{m2}+sL_{m2}\right)}{R_{m1}R_{m2}+sR_{m1}L_{m2}+\left(R_{m0}+sL_{m1}\right)\left(R_{m1}+R_{m2}+sL_{m2}\right)}=\frac{\Gamma (s)}{\Upsilon (s)},$$

with deg(Γ(s))=1 and deg(Υ(s))=2, resulting into a transfer function consisting of one zero and two poles. By inserting the transfer function $\frac{\Psi_{tot}\left(s\right)}{I_{s}\left(s\right)}$ into Equation (13), the transfer function of the current can be expressed as:

(16) $$\frac{I_{s}\left(s\right)}{U(s)}=\frac{\Upsilon (s)}{R_{s}\Upsilon \left(s\right)+s\Gamma \left(s\right)}=\frac{\Xi (s)}{\Lambda (s)}\;\mathrm{with}\;\deg\left(\Xi \left(s\right)\right)=2\;\mathrm{and}\;\deg\left(\Lambda \left(s\right)\right)=2.$$

Similarly to the case N=1, a zero gets added to the magnetic transfer function when it is expressed in electrical terms. Generally, adding a RL element to the cascade shown in Figure 2 adds a pole and a zero to the transfer function of the current, as it can be seen from Equation (16). Given the nature of the magnetic RL circuits, they can be considered as low pass filters for the magnetic flux, which are delaying the rise of the magnetic flux. Transformed to the electrical circuit, they represent high pass filters for the resulting electrical current. This is due to the fact that the delayed rise of flux causes a delayed rise of the electrical inductance, therefore allowing the electrical current to rise faster until all eddy currents are vanished. Similar results are observed in [31].

**Figure 3:**
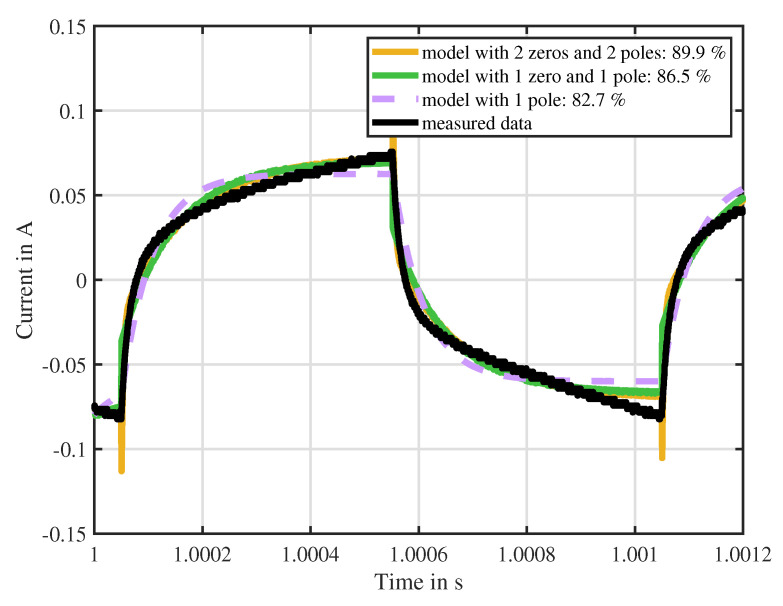
Approximation of the current ripple. Comparison between measured current ripple of the actuator ITS-LZ 1949 from Red Magnetics and the proposed models with different model complexity. Model accuracy is shown in percentage in comparison to the measured data.

In order to simplify the mathematical treatment of the eddy currents and reduce the number of parameters in this model, it is desired to reduce the number *N* of eddy current elements. In the following, an experimental measurement of the current ripple of the actuator under test ITS-LZ 1949 from Red Magnetics is shown and compared to the discussed models. This experimental result is already anticipated for this discussion, while the solenoid actuator itself, its parameters and the test-bench are described in detail in Section 4. Figure 3 shows the performance of the models with different model complexity. Firstly, a current transfer function with no zeros and one pole is evaluated. This represents the case where no eddy currents are present since $L_{m1}$ equals zero. Secondly, a transfer function consisting of one zero and one pole, like Equation (14), is considered. Finally, a transfer function with two zeros and two poles, as indicated in Equation (16), is shown. From the experimental data it can be seen that the switching PWM voltage causes a considerable cusp in the current response especially at the switching time instants. This cusp vanishes over time, resulting into the classical exponential behavior of the current ripple of an electromagnetic actuator. The model without eddy currents lacks in estimation performance since it cannot approximate the cusp. Compared to this, the model with one pole and one zero represents a better approximation with 86.5%. Instead, the model containing two poles and two zeros shows a good performance but has undesired overshoot at the switching time instants. Due to its good estimation behavior and its simplicity, the model with one eddy current element (N=1) will be considered from now on. Indeed, the work [32] also uses such a reduced eddy current model, which introduces instantaneous cusps at the switching instants.

**Figure 4:**
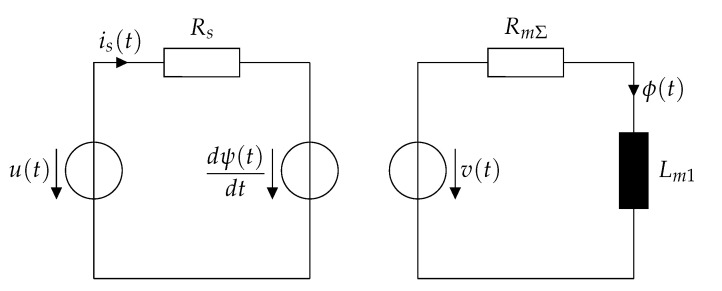
Electromagnetic equivalent circuit of a solenoid actuator considering only the dominant eddy current branch.

The reluctances in the model can be summarized into one reluctance denoted as $R_{m\Sigma }$:

(17) $$R_{m\Sigma }=R_{m0}+R_{m1},$$

leading to the equivalent circuit illustrated in Figure 4.

The calculations made above are conducted in the Laplace domain for sake of comprehension. Nevertheless, the parameters $R_{m\Sigma }\left(x\right)$ and $L_{m1}\left(x\right)$ are position dependent. Since the position changes during operation of the actuator, also the time derivatives of these parameters need to be respected since they induce voltages inside the actuator. Therefore, the following analysis will take place in the time domain. The magnetic circuit can be modeled as follows:

(18) $$\begin{array}{cc} v(t) & =R_{m\Sigma }\left(x\right)\phi \left(t\right)+L_{m1}\left(x\right)\frac{\partial \phi (t)}{\partial t}, \end{array}$$

(19) $$\begin{array}{cc} W^{2}i_{s}\left(t\right) & =R_{m\Sigma }\left(x\right)\psi \left(t\right)+L_{m1}\left(x\right)\frac{\partial \psi (t)}{\partial t}, \end{array}$$

while the electrical circuit can be described as:

(20) $$u\left(t\right)=R_{s}i_{s}\left(t\right)+\frac{\partial \psi (t)}{\partial t},$$

and can be transformed into:

(21) $$\psi \left(t\right)=\int \left(u\left(t\right)-R_{s}i_{s}\left(t\right)\right)dt.$$

By means of algebraic manipulation, Equations (19)–(21) can be expressed as:

(22) $$i_{s}\left(t\right)=\frac{R_{m\Sigma }\left(x\right)}{W^{2}}\left(\int \left(u\left(t\right)-R_{s}i_{s}\left(t\right)\right)dt\right)+\frac{L_{m1}\left(x\right)}{W^{2}}\left(u\left(t\right)-R_{s}i_{s}\left(t\right)\right).$$

The term $\frac{W^{2}}{R_{m\Sigma }\left(x\right)}$ represents the definition of an electrical inductance and will be from now on indicated as differential inductance $L_{d}\left(x\right)$. The term differential is chosen here since the actuator is driven by a PWM voltage which excites the inductance in the small signal range. The term $\frac{W^{2}}{L_{m1}\left(x\right)}$ represents the inverse of a resistance, which is defined as parallel resistance $R_{p}\left(x\right)$. This resistor incorporates the eddy current behavior. Rearranging the equation:

(23) $$L_{d}\left(x\right)i_{s}\left(t\right)=\int \left(u\left(t\right)-R_{s}i_{s}\left(t\right)\right)dt+\frac{L_{d}\left(x\right)}{R_{p}\left(x\right)}\left(u\left(t\right)-R_{s}i_{s}\left(t\right)\right),$$

and differentiating it under consideration of all time-dependent parameters, yields to:

(24) $$\begin{array}{ll} \frac{\partial L_{d}\left(x\right)}{\partial x}\frac{\partial x}{\partial t}i_{s}\left(t\right)+L_{d}\left(x\right)\frac{\partial i_{s}\left(t\right)}{\partial t} & =u\left(t\right)-R_{s}i_{s}\left(t\right)+\frac{\partial }{\partial x}\left(\frac{L_{d}\left(x\right)}{R_{p}\left(x\right)}\right)\frac{\partial x}{\partial t}\left(u\left(t\right)-R_{s}i_{s}\left(t\right)\right)\\ & +\frac{L_{d}\left(x\right)}{R_{p}\left(x\right)}\left(\frac{u(t)}{\partial t}-R_{s}\frac{\partial i_{s}\left(t\right)}{\partial t}\right). \end{array}$$

Reformulating this equation leads to a classical differential equation with the input u(t) and the output $i_{s}\left(t\right)$:

(25) $$\begin{array}{c} \left(1+\frac{\partial }{\partial x}\left(\frac{L_{d}\left(x\right)}{R_{p}\left(x\right)}\right)\frac{\partial x}{\partial t}\right)u\left(t\right)+\frac{L_{d}\left(x\right)}{R_{p}\left(x\right)}\frac{\partial u(t)}{\partial t}=\left(R_{s}+\frac{\partial L_{d}\left(x\right)}{\partial x}\frac{\partial x}{\partial t}+R_{s}\frac{\partial }{\partial x}\left(\frac{L_{d}\left(x\right)}{R_{p}\left(x\right)}\right)\frac{\partial x}{\partial t}\right)i_{s}\left(t\right)\\ +L_{d}\left(x\right)\left(1+\frac{R_{s}}{R_{p}\left(x\right)}\right)\frac{\partial i_{s}\left(t\right)}{\partial t} \end{array}.$$

In common solenoid actuators, the parallel resistance $R_{p}$ is usually several orders of magnitude higher than the inductance $L_{d}$, since $R_{p}$ only resembles a leakage component in parallel to the main inductor. The experimental results presented in Section 4 verify this consideration. Thus, the back-induced component $\frac{\partial }{\partial x}\left(\frac{L_{d}\left(x\right)}{R_{p}\left(x\right)}\right)\frac{\partial x}{\partial t}$ can be considered much smaller than 1. Under this assumption, the equation can be simplified to:

(26) $$u\left(t\right)+\frac{L_{d}\left(x\right)}{R_{p}\left(x\right)}\frac{\partial u(t)}{\partial t}=R_{\Sigma }i_{s}\left(t\right)+L_{d}\left(x\right)\left(1+\frac{R_{\Sigma }}{R_{p}\left(x\right)}\right)\frac{\partial i_{s}\left(t\right)}{\partial t},$$

with the total resistance $R_{\Sigma }$, consisting of the series resistance and the back-induced components, being defined as:

(27) $$R_{\Sigma }=R_{s}+\frac{\partial L_{d}\left(x\right)}{\partial x}\frac{\partial x}{\partial t}+R_{s}\frac{\partial }{\partial x}\left(\frac{L_{d}\left(x\right)}{R_{p}\left(x\right)}\right)\frac{\partial x}{\partial t}.$$

**Figure 5:**
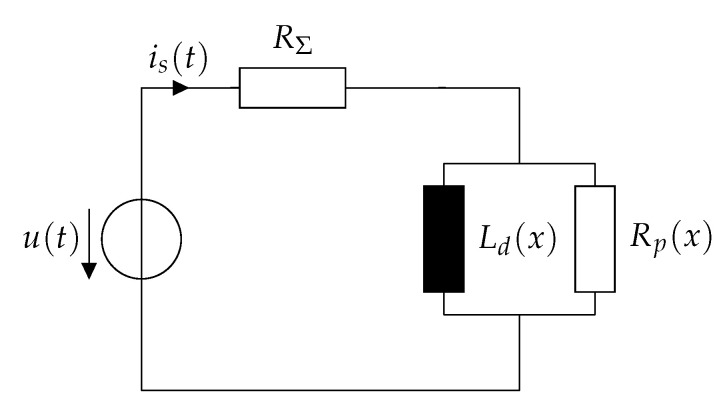
Simplified electrical equivalent circuit including the eddy current effects.

Equation (26) resembles the differential equation of the electrical equivalent circuit that is shown in Figure 5. Such a circuit including a parallel resistance which accounts for losses is common in many works such as [23,26,29]. Unlike the work [26], parasitic capacitances are not considered since they complicate the mathematical treatment significantly while their influence on the current response is negligible [23,26,33]. The connection between this circuit and the differential equation is analyzed thoroughly in [26].

### 2.3. Current Ripples Induced by a PWM Voltage

Electromagnetic actuators are usually driven by PWM-based switching power electronics. In this work, a bipolar edge-aligned PWM is considered that can be formulated as

(28) $$u_{pwm}\left(t\right)=\left\{\begin{array}{c} +U_{DC}\;\mathrm{for}\;0\leq t\leq \alpha \cdot t_{pwm}\\ -U_{DC}\;\mathrm{for}\;\alpha \cdot t_{pwm}\leq t\leq t_{pwm} \end{array}\right.,$$

with $U_{DC}$ being the DC link voltage, $t_{pwm}$ being the PWM period and α being the duty cycle ranging from 0% to 100%. In particular, this equation contains discontinuities at the switching instants, complicating the closed analytical solution of Equation (26), because a derivative of the input is needed. Therefore, in order to model correctly those discontinuities, the Heaviside step function is considered. For sake of brevity and comprehension, only the rising edge of the edge-aligned PWM pulse will be discussed from now on. Unlike the falling edge, this edge does not depend on the actual value of the duty cycle α and therefore occurs always at a fixed timing. Expressions for the falling edge and for non-edge-aligned PWM patterns can be derived in a similar manner.

The rising edge of the PWM voltage can be formulated as:

(29) $$u\left(t\right)=-U_{DC}+2U_{DC}\Theta \left(t\right),$$

with the Heaviside step function Θ(t) being defined as:

(30) $$\Theta \left(t\right)=\left\{\begin{array}{c} 0\;\mathrm{for}\;t<0\\ 1\;\mathrm{for}\;t\geq 0 \end{array}\right..$$

Solving the differential Equation (26) for the input given by Equation (29) yields to

(31) $$i_{s}\left(t\right)=\left\{\begin{array}{c} e^{\left(-\frac{t}{\tau_{el}}\right)}\left(i_{0}+\frac{U_{DC}}{R_{\Sigma }}-\frac{2U_{DC}}{R_{\Sigma }+R_{p}}\right)-\frac{U_{DC}}{R_{\Sigma }}\;\mathrm{for}\;t<0\\ e^{\left(-\frac{t}{\tau_{el}}\right)}\left(i_{0}+\frac{U_{DC}}{R_{\Sigma }}-\frac{2U_{DC}}{R_{\Sigma }+R_{p}}\right)-\frac{U_{DC}}{R_{\Sigma }}+\frac{2U_{DC}}{R_{\Sigma }}\left(1-e^{\left(-\frac{t}{\tau_{el}}\right)}\right)+\frac{2U_{DC}}{R_{\Sigma }+R_{p}}e^{\left(-\frac{t}{\tau_{el}}\right)}\;\mathrm{for}\;t\geq 0 \end{array}\right.,$$

with the electrical time constant $\tau_{el}$ being

(32) $$\tau_{el}=\frac{L_{d}\left(R_{p}+R_{\Sigma }\right)}{R_{p}R_{\Sigma }}.$$

**Figure 6:**
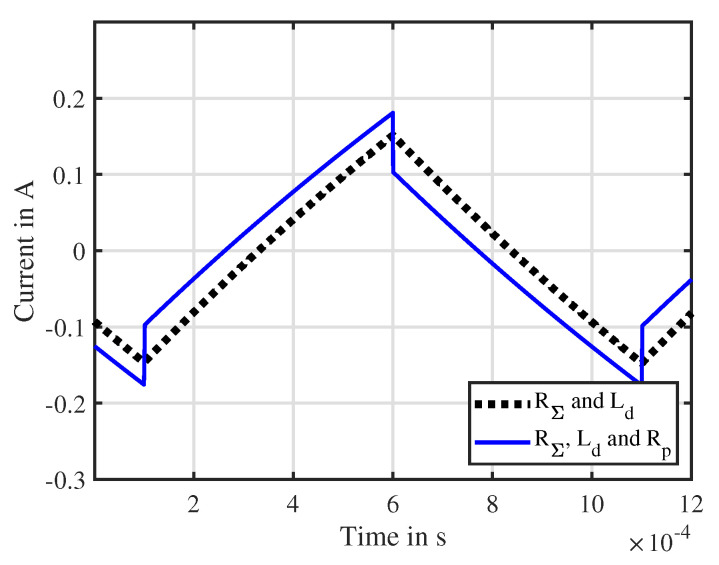
Simulated response of the current $i_{s}\left(t\right)$ in the electrical equivalent circuit when a pulse width modulated (PWM) voltage is applied in comparison to a classical RL circuit for the parameters $U_{DC}=12$ V, $R_{\Sigma }=10$ Ω, $R_{p}=300$ Ω and $L_{d}=20$ mH.

This result considers the parameters to be constant over one PWM period, which simplifies the analytical solution. Due to the fact that the mechanical time constant of the actuator is considerable larger than the PWM period, this assumption can be applied. Figure 6 illustrates the response of the current to a PWM voltage for two cases: an actuator without eddy currents (black) and an actuator with eddy currents (blue). In case no eddy currents are present, the response is the classical ripple which has been previously described in the work [26]. Under consideration of eddy currents, cusps in the current response appear. These cusps occur at the switching time instant of the PWM voltage and change in their sign accordingly if a rising or falling edge is active. Because only one RL element is modeled, those cusps happen instantaneously, which is physically not feasible. As discussed above, increasing the model order by increasing the number *N* of RL elements smooths that current jump. Nevertheless, the height of that cusp can be modeled appropriately even with a number N=1, therefore this model is preferred for the identification of $R_{p}$.

## 3. Position Estimation Using Differential Inductance and Eddy Current Information

In the following, position estimation techniques are presented and an approach for data fusion of different position estimates in terms of a multilayer perceptron neural network is discussed. The first estimator is based on the identification of the differential inductance $L_{d}$, a parameter that has high sensitivity towards position but exhibits an ambiguity in the estimated value. The second estimator exploits the knowledge of the parallel resistor $R_{p}$, which offers a unique estimate but with poor sensitivity and SNR.

### 3.1. Inductance-Based Position Estimation

**Figure 7:**
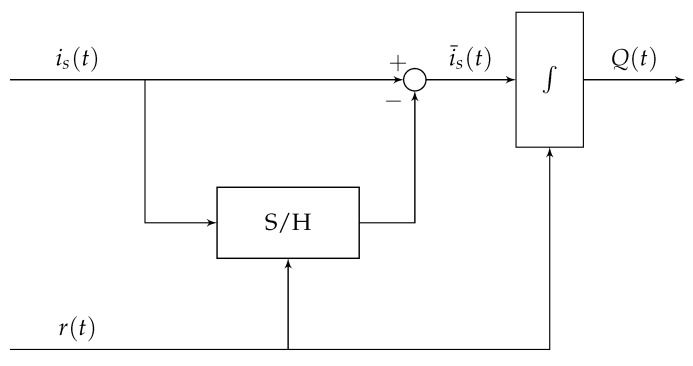
Schematic used for the implementation of the IDIM technique, including an offset-eliminating stage and an analog integrator with reset capability, adopted from [26].

In the following section, the Integrator-Based Direct Inductance Measurement (IDIM) technique is described briefly, which is based on the analog integration of the current ripple in order to estimate the differential inductance of a solenoid actuator. The technique is explained, derived and validated thoroughly in [25,26] and, for further information, reference is made to those works. Figure 7 shows the analog circuitry which is suitable for the implementation of the IDIM technique. Its first stage is an offset-eliminating stage that samples and removes the fundamental current component $i_{s}\left(0\right)$ by using a sample and hold stage (S/H) and a subtracting amplifier. The following stage is an analog integrator that can be externally reset with a trigger signal denoted as r(t). The concept of analog integration avoids oversampled current measurements and increases the signal-to-noise ratio. By applying the external reset at every PWM time period, a drift of the integrator can be avoided.

The reset trigger signal r(t) can be chosen as [26]:

(33) $$r\left(t\right)=\left\{\begin{array}{c} 0\;\mathrm{for}\;t_{s}^{+}\leq t\leq t_{e}^{+}\\ 0\;\mathrm{for}\;t_{s}^{-}\leq t\leq t_{e}^{-}\\ 1\;\mathrm{else} \end{array}\right.,$$

with the times being

(34) $$t_{s}^{+}=t_{r},\;t_{e}^{+}=\alpha \cdot t_{pwm},\;t_{s}^{-}=\alpha \cdot t_{pwm}+t_{r},\;t_{e}^{-}=t_{pwm}-t_{r}.$$

**Figure 8:**
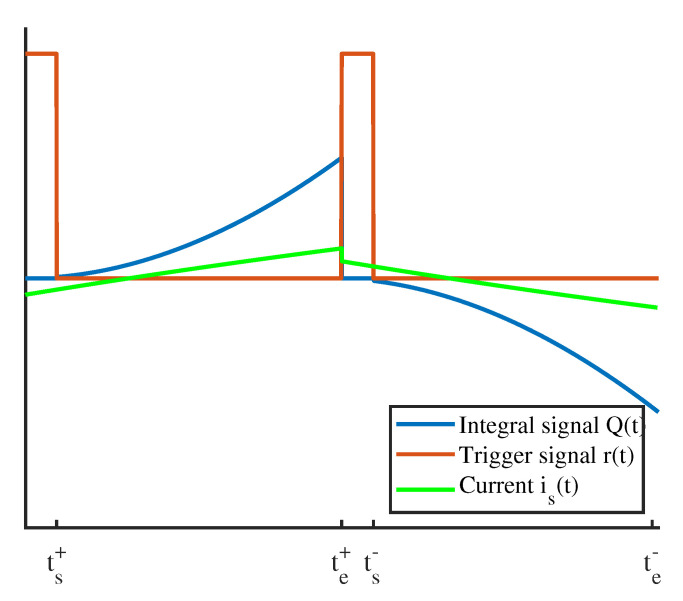
Current $i_{s}\left(t\right)$, Integral Q(t) and corresponding reset trigger r(t) used for the estimation of the differential inductance $L_{d}$ by means of the Integrator-Based Direct Inductance Measurement (IDIM) technique.

The trigger signal is designed in such a way that PWM switching instants are avoided during integration. In particular, the waiting time $t_{r}$ after each switching instant is a design parameter that must be dimensioned in such a way that the integration takes place only when the eddy currents are vanished. Therefore, their influence on the inductance estimation can be minimized significantly. Other techniques, which are based on the estimation of the differential inductance, avoid these time instants during the estimation procedure [10,21,22]. The current ripple that is being integrated, the corresponding output of the integrator Q(t) and the reset trigger signal r(t) are shown in Figure 8.

The behavior of the analog signal-processing circuit shown in Figure 7 can be mathematically expressed as:

(35) $$Q\left(t\right)=\int_{r(t)\neq 1}\left(i_{s}\left(t\right)-i_{s}\left(t_{x}\right)\right)dt,$$

where $t_{x}$ stands either for the time $t_{s}^{+}$ when the positive voltage pulse is considered and $t_{s}^{-}$ when the negative voltage pulse is considered. Since the time-integral of a current resembles physically an electrical charge, the integral is denoted here as Q(t). Nevertheless, in practical applications such as in the case of a shunt-based current sensor, the sensed current is usually measured as an analog voltage, which is proportional to the current. Thus, Q(t) is measured as an analog voltage.

Inserting Equation (26) into the Equation (35) of integral Q(t) yields to:

(36) $$Q\left(t\right)=\int_{r(t)\neq 1}\left[\frac{1}{R_{\Sigma }}\left(u\left(t\right)+\frac{L_{d}}{R_{p}}\frac{\partial u(t)}{\partial t}-L_{d}\left(1+\frac{R_{s}}{R_{p}}\right)\frac{\partial i_{s}\left(t\right)}{\partial t}\right)-i_{s}\left(t_{x}\right)\right]dt.$$

The integral can be solved for the PWM input $u_{pwm}\left(t\right)$ expressed in Equation (28) and can be simplified under the assumption $R_{s}<<R_{p}$, since the series resistance $R_{s}$ is usually designed to be very small compared to the parallel resistance [26]. For most actuators, the mechanical time constant is significantly larger than the electrical time constant and the PWM time period. During integration, it is therefore assumed that the position-dependent parameters stay constant over one PWM period. For the positive voltage pulse, the integral can be obtained as [26]:

(37) $$Q\left(t_{e}^{+}\right)\approx \frac{U_{DC}}{R_{\Sigma }}\left(t_{e}^{+}-t_{s}^{+}\right)-\frac{L_{d}}{R_{\Sigma }}\left(i_{s}\left(t_{e}^{+}\right)-i_{s}\left(t_{s}^{+}\right)\right)-i_{s}\left(t_{s}^{+}\right)\left(t_{e}^{+}-t_{s}^{+}\right).$$

In a similar way, the integral of the negative pulse can be obtained [26]:

(38) $$Q\left(t_{e}^{-}\right)\approx \frac{-U_{DC}}{R_{\Sigma }}\left(t_{e}^{-}-t_{s}^{-}\right)-\frac{L_{d}}{R_{\Sigma }}\left(i_{s}\left(t_{e}^{-}\right)-i_{s}\left(t_{s}^{-}\right)\right)-i_{s}\left(t_{s}^{-}\right)\left(t_{e}^{-}-t_{s}^{-}\right).$$

Both equations can be merged into a matricial form [26]:

(39) $$\left[\begin{array}{c} U_{DC}\left(t_{e}^{+}-t_{s}^{+}\right)\\ -U_{DC}\left(t_{e}^{-}-t_{s}^{-}\right) \end{array}\right]\approx A\left[\begin{array}{c} R_{\Sigma }\\ L_{d} \end{array}\right],$$

with the matrix **A** being [26]:

(40) $$A=\left[\begin{array}{cc} Q\left(t_{e}^{+}\right)+i_{s}\left(t_{s}^{+}\right)\left(t_{e}^{+}-t_{s}^{+}\right) & i_{s}\left(t_{e}^{+}\right)-i_{s}\left(t_{s}^{+}\right)\\ Q\left(t_{e}^{-}\right)+i_{s}\left(t_{s}^{-}\right)\left(t_{e}^{-}-t_{s}^{-}\right) & i_{s}\left(t_{e}^{-}\right)-i_{s}\left(t_{s}^{-}\right) \end{array}\right].$$

By solving the system of linear equations, the differential inductance can be estimated [26]:

(41) $$L_{d}\approx \frac{-U_{DC}}{\left|A\right|}\left[\left(Q\left(t_{e}^{-}\right)+i_{s}\left(t_{s}^{-}\right)\left(t_{e}^{-}-t_{s}^{-}\right)\right]\left(t_{e}^{+}-t_{s}^{+}\right)+\left[Q\left(t_{e}^{+}\right)+i_{s}\left(t_{s}^{+}\right)\left(t_{e}^{+}-t_{s}^{+}\right)\right]\left(t_{e}^{-}-t_{s}^{-}\right)\right),$$

with |A| being the determinant of the matrix **A**.

### 3.2. Eddy Current-Based Position Estimation

In order to solve the ambiguities that occur when using approaches based on the differential inductance, another parameter needs to be determined which allows a unique estimation of the actuator position. As seen in Section 2, the eddy currents exhibit a significant dependence on the position. Those eddy currents can be modeled by means of lumped magnetic inductances. In particular, voluminous pieces of magnetic material such as the plunger can be represented by a cascade of magnetic RL-circuits in order to model the skin effect. Increasing the number *N* of RL elements also increases the accuracy of the model, while increasing the modeling and computational effort. As mentioned in Section 2, assuming only one eddy current element (N=1) with one lumped magnetic inductance allows for a sufficient estimation accuracy of the current ripple, especially at the cusp, while minimizing the modeling effort significantly. Using only one lumped magnetic inductance neglects the presence of the skin effect, therefore the cusp in the current ripple occurs as a discontinuity. Nevertheless, the cusp needs physically a certain time to decay. Using one magnetic inductance in the magnetic circuit result into a parallel resistance $R_{p}$ in the electrical circuit, as shown in Figure 5. In the following, a measurement procedure for this resistor is presented with the aim of a unique estimate of the position through this quantity.

**Figure 9:**
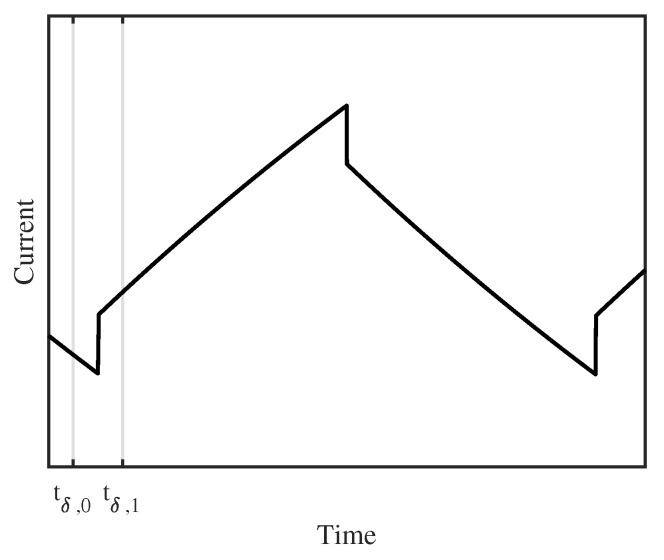
Current ripple with the proposed approximation of the eddy currents. The timings for the identification of the parallel resistance $R_{p}$ are indicated as $t_{\delta ,0}$ and $t_{\delta ,1}$.

From Equation (31), it can be seen that the switching of the PWM voltage induces a cusp into the current response. The height of that cusp can be calculated by the difference of the piece-wise defined Equation (31) between the two cases t<0 and t≥0. In the presented model, this cusp is a discontinuity and needs no time to decay, while eddy currents need a certain time to fade out in practical applications. Therefore, the measurement of the cusp height is conducted at two measurement points. Those points should be theoretically as close as possible to the switching time instant, while, practically, a certain waiting time needs to be applied to ensure that the eddy currents are decayed. The first measurement point $t_{\delta ,0}$ is placed before the cusp, the second point $t_{\delta ,1}$ afterwards, as illustrated in Figure 9. Mathematically, it can be written:

(42) $$\begin{array}{cc} t_{\delta ,0} & <0, \end{array}$$

(43) $$\begin{array}{cc} t_{\delta ,1} & \geq 0. \end{array}$$

The height of the cusp can be calculated with Equation (31) as:

(44) $$\begin{array}{c} \Delta i_{s}\left(t\right)=i_{s}\left(t_{\delta ,1}\right)-i_{s}\left(t_{\delta ,0}\right)=e^{\left(-\frac{t_{\delta ,1}}{\tau_{el}}\right)}\left(i_{0}+\frac{U_{DC}}{R_{\Sigma }}-\frac{2U_{DC}}{R_{\Sigma }+R_{p}}\right)+\frac{2U_{DC}}{R_{\Sigma }}\left(1-e^{\left(-\frac{t_{\delta ,1}}{\tau_{el}}\right)}\right)\\ +\frac{2U_{DC}}{R_{\Sigma }+R_{p}}e^{\left(-\frac{t_{\delta ,1}}{\tau_{el}}\right)}-e^{\left(-\frac{t_{\delta ,0}}{\tau_{el}}\right)}\left(i_{0}+\frac{U_{DC}}{R_{\Sigma }}-\frac{2U_{DC}}{R_{\Sigma }+R_{p}}\right). \end{array}$$

By considering the measurements time instants $t_{\delta ,0}$ and $t_{\delta ,1}$ significantly smaller than the PWM time period, it can be assumed that:

(45) $$\begin{array}{c} t_{\delta ,0}\to 0^{-}, \end{array}$$

(46) $$\begin{array}{c} t_{\delta ,1}\to 0^{+}. \end{array}$$

Therefore, the height of the cusp can be approximated as:

(47) $$\tilde{\Delta i_{s}}\approx \lim_{\begin{array}{c} t_{\delta ,0}\to 0^{-}\\ t_{\delta ,1}\to 0^{+} \end{array}}\Delta i_{s}\left(t_{\delta ,0},t_{\delta ,1}\right)=\frac{2U_{DC}}{R_{\Sigma }+R_{p}}.$$

Finally, an estimate of the position-dependent parallel resistance can be obtained:

(48) $$R_{p}\approx \frac{2U_{DC}}{\tilde{\Delta i_{s}}}-R_{\Sigma }.$$

It has to be highlighted that the estimation of the parallel resistance depends on the difference between two current samples, which are very close to each other. Measurement noise as well as limited bandwidth and slew rate of the current sensor have a significant influence of the obtained estimate. It is therefore desired to use a high bandwidth current sensor with anti-aliasing filter in front of the AD converter. Additionally, in order to increase the SNR, the estimate of the resistance needs to be low pass filtered digitally with a cut-off frequency that is higher than the mechanical frequency of the actuator.

### 3.3. Position Data Fusion

In Section 3.1 and Section 3.2, two estimation approaches are proposed for identifying the differential inductance $L_{d}$ and the parallel resistance $R_{p}$ of the solenoid actuator under operation. As already mentioned above, the differential inductance $L_{d}$ exhibits a remarkable dependency on the actuator position $L_{d}=f\left(x\right)$. Nevertheless, its characteristic is not bijective, which means that a global inverse $x=f^{-1}\left(L_{d}\right)$ does not exist. This leads to ambiguities in the position estimate. Nevertheless, the parallel resistance has a bijective characteristic over the entire position range, which ensures the global invertability of the function $R_{p}=f\left(x\right)\to x=f^{-1}\left(R_{p}\right)$, thus allowing a unique estimation of the position [23,27,28].

Despite the problem of ambiguities, the position estimation through the differential inductance $L_{d}$ is usually preferred in the state-of-the-art works, since this parameter offers a high sensitivity. Works describing the behavior of the parallel resistance $R_{p}$ concede that this parameter is usually strongly affected by a large variance [23]. Given the mentioned disadvantages of both approaches, it is desired to merge both information into one position estimate. By using the parallel resistance information for a rough estimate and the inductance for a precise estimate, it is possible to combine the advantages of both approaches, namely the uniqueness and the precision. In literature, this has been conducted mainly by binary decision-making based on the knowledge of $R_{p}$ [28]. This simple solution offers already a good approximation, but can fail e.g., when noise in the estimate of $R_{p}$ triggers the binary rule.

Instead, this work uses a multilayer perceptron (MLP) neural network for data fusion. Such an MLP can train its input weights based on experimental data, that allows the MLP to give more weight on the input $L_{d}$ for ensuring a precise estimate with high SNR and less weight on $R_{p}$, which is in this case only used as support information for finding a unique estimate. Moreover, neural networks have proven to be nonlinear interpolators with good interpolation and extrapolation capability [34], thus allowing a good identification of nonlinear mappings such as the characteristic of a solenoid actuator [35]. The implementation of the MLP on the actuator under test is shown in Section 4.

**Figure 10:**
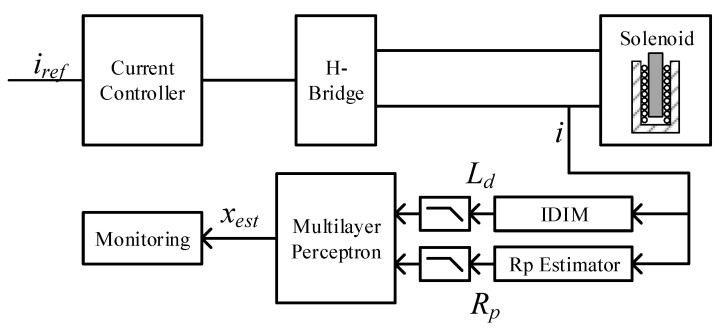
Schematic of the position estimation algorithm together with current controller, H-bridge and solenoid actuator.

Figure 10 summarizes the signal measurement and processing chain, where the solenoid actuator is driven with a PWM switching voltage and the resulting current is measured. Based on that measured current, the identification of the electrical parameters $L_{d}$ and $R_{p}$ is conducted separately. Both information are filtered digitally with low pass filters, that should be designed with the same cutoff frequency in order to avoid phase shifts between both signals. The MLP estimates a unique position information out of both parameters. That estimated position can be used for various monitoring or control purposes, such as the end-position detection, which is described in Section 4. Additionally, a current controller ensures a constant driving current in the coil even under changing DC link voltage or resistance due to self-heating.

## 4. Experimental Results

In the following section, experimental results of the proposed position estimator based on the identified differential inductance $L_{d}$ and parallel resistance $R_{p}$ are shown and discussed. Experimental validation is preferred over numerical validation due to the complicity of a numerical simulation. Since the relative magnetic permeability is working-point dependent and exhibits a hysteretic behavior, finite element methods (FEMs) have to be applied taking also into account the presence of eddy currents. Moreover, accurate geometries and material data are required for a precise numerical result.

**Figure 11:**
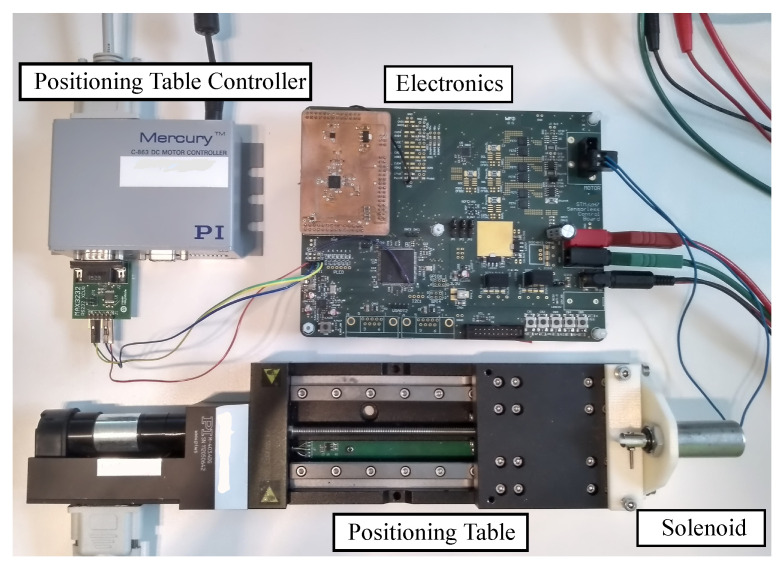
Test-bench used for characterization and validation of the solenoid actuator, including linear positioning stage, dedicated electronics as well as solenoid actuator under test.

The experiments were obtained by means of the test-bench shown in Figure 11, which consisted of custom electronics developed at the Laboratory of Actuation Technology, a linear high-precision positioning table as well as the solenoid actuator under test. The custom electronics contained an H-Bridge capable of driving the solenoid actuator, a STM32H7 microcontroller, 16 bit AD converters and the analog IDIM circuit shown in Figure 7. The current was measured with an AD8418 shunt amplifier from Analog Devices in the phase of the solenoid actuator with a bandwidth of 250 kHz [36].

The linear positioning table M403.4DG from Physics Instruments was operated under position control with 200 nm resolution and was able to block the position with forces up to 50 N [37]. The table was connected mechanically to the solenoid actuator ITS-LZ 1949 from Red Magnetics [38], which was used for identification and validation of the shown approach. The actuator was driven with a voltage of 24 V. The parameters of this solenoid actuator as well as the corresponding parameters of the IDIM technique and the parallel resistance estimator are listed in Table 1.

**Figure 12:**
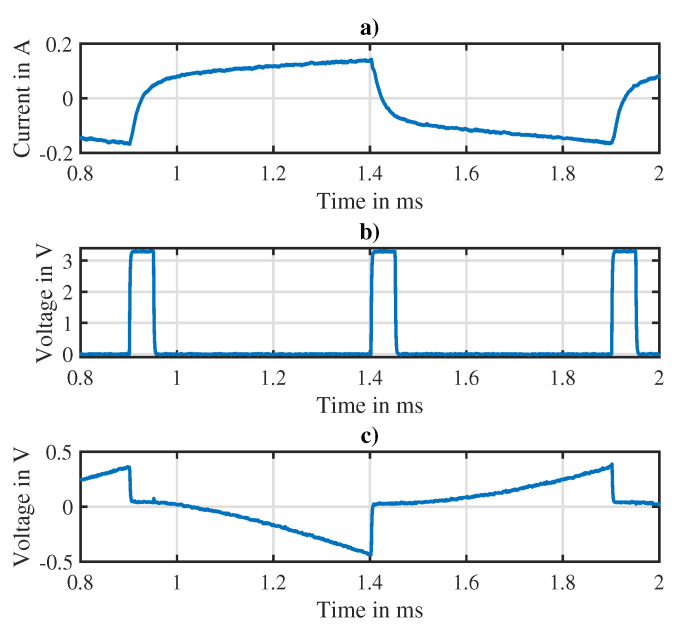
Experimental measurements, (**a**) current ripple induced by the bipolar PWM voltage, (**b**) trigger signal necessary for the IDIM implementation, (**c**) corresponding output of the analog integrator.

The PWM frequency of the actuator was chosen to be 1 kHz in order to avoid a decrease of the differential inductance present at higher frequencies. Figure 12 shows the resulting current ripple as well as the trigger signal r(t) of the IDIM technique and the corresponding output Q(t) of the integrator stage. The cusps in the measured current are clearly visible and predominate the current ripple. As mentioned in Section 3.1, the trigger signal of the IDIM technique was chosen in such a way that those cusps were avoided during integration.

### 4.1. Characterization of the Actuator

In order to estimate the position based on the determined values of $L_{d}$ and $R_{p}$, the dependency of those parameters on the position needs to be identified. Therefore, the test-bench with its position-controlled linear table was used that allowed us to block the solenoid plunger at a certain position while different controlled currents are applied. Thus, a mapping of the parameters over the entire current and position range can be obtained experimentally. Per each current-position pair, the identified values of $L_{d}$ and $R_{p}$ were averaged over 1000 samples.

**Figure 13:**
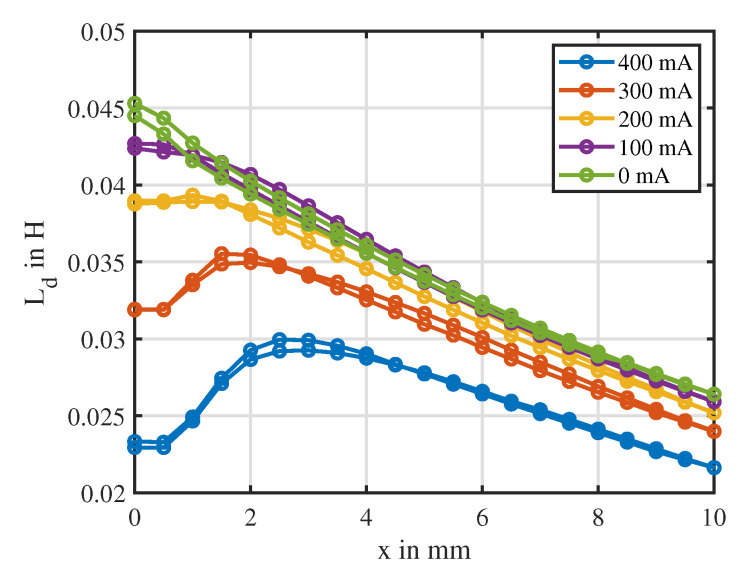
Characteristic of the differential inductance over the position at different fixed currents. Measurement points are indicated by dotted points.

Figure 13 shows the dependency of the differential inductance $L_{d}$ on the position at different fixed currents. It can be seen that the differential inductance at zero current had a hyperbolic behavior with a slight hysteresis over the position. With increasing current, the inductance decreased due to magnetic saturation [29] and a current-dependent extremum occurred at a certain position value. Due to these extrema, the position estimation based on the differential inductance $L_{d}$ suffered from ambiguities and from little sensitivity at the extremum itself. All curves exhibited a slight hysteretic behavior over the position, which was neglected during the following work.

**Figure 14:**
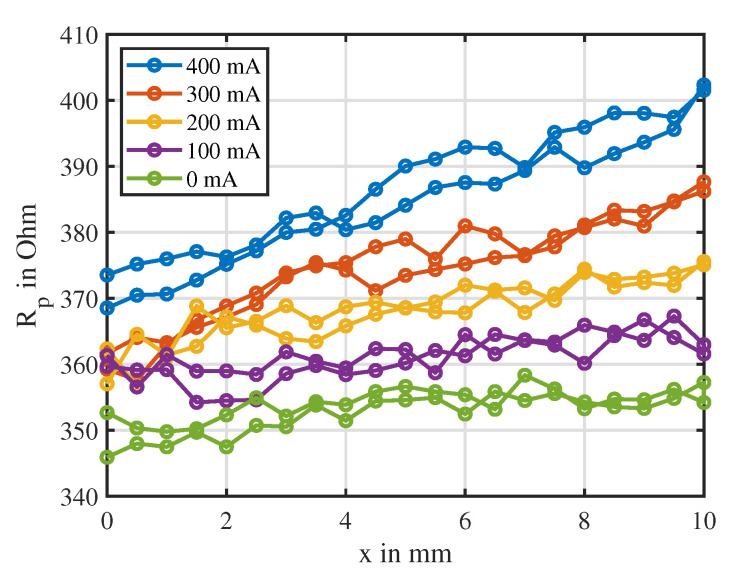
Characteristic of the parallel resistance over the position at different fixed currents. Measurement points are indicated by dotted points.

Figure 14 illustrates the identified characteristic of the parallel resistance $R_{p}$ over the position at different currents. All curves showed a nearly linear dependency on the position. The offsets and the slopes of these linear curves increased with rising current. Over the entire position and current range, no extrema and therefore no ambiguities occurred, thus a unique estimate of the position was feasible. Nevertheless, all measurements showed a large variance. This resembles the observation from [23], where a large deviation of the $R_{p}$ estimate was also determined. Similarly to the estimate of $L_{d}$, the identified curves showed hysteresis, which was neglected in this work. It has to be denoted that the value of $R_{p}$ was significantly temperature-dependent, since its value was based on the magnetic inductance. This parameter depended on the temperature-sensitive material conductivity σ, as visible in Equation (3). Thus, self-heating of the actuator needs to be taken into account.

This work evaluated the position estimation on a use case scenario involving end-position detection for a switching solenoid actuator. In such a use case, only two current conditions, in particular zero mean current and full mean current ($i_{s}$ = 400 mA), were of interest for the position estimator. In the case of the curve representing the zero current condition, no ambiguity was visible and therefore a polynomial model, which was only based on the differential inductance $L_{d}$, could be used for position estimation [26]. In particular, a 2nd order polynomial model was chosen. In the case of the actuator under full mean current, there existed an extremum at a position of approx. 2.5 mm and the data fusion approach mentioned in Section 3.3 based on an MLP neural network for the inputs $L_{d}$ and $R_{p}$ could be applied. Particularly, the MLP used in this work consisted of one hidden layer with 15 neurons with sigmoidal activation functions and one output neuron for the estimated position. The training of the MLP was made by the Bayesian Regularization back-propagation algorithm. During the fitting process of the polynomial model and the neural network, the hysteresis was neglected by assuming a middle curve, which was placed between the forward direction curve and the backwards direction curve. Since the MLP neural network contained only one hidden layer with a small number of neurons, it could be implemented even on microcontrollers with low computational power.

### 4.2. End-Position Detection

The proposed position estimator was validated in the use case scenario of an end-position detection for switching solenoid actuators. During this experiment, the actuator was either in zero mean current condition or in full mean current condition. The positioning table was able to move the plunger in a quasi-static condition over the entire position range and the position measured by the positioning table was compared to the position estimated by the discussed approach. The experiments were conducted in a short time so that the effect of temperature rise due to self-heating of the actuator could be considered small given the large thermal time constant of the system.

**Figure 15:**
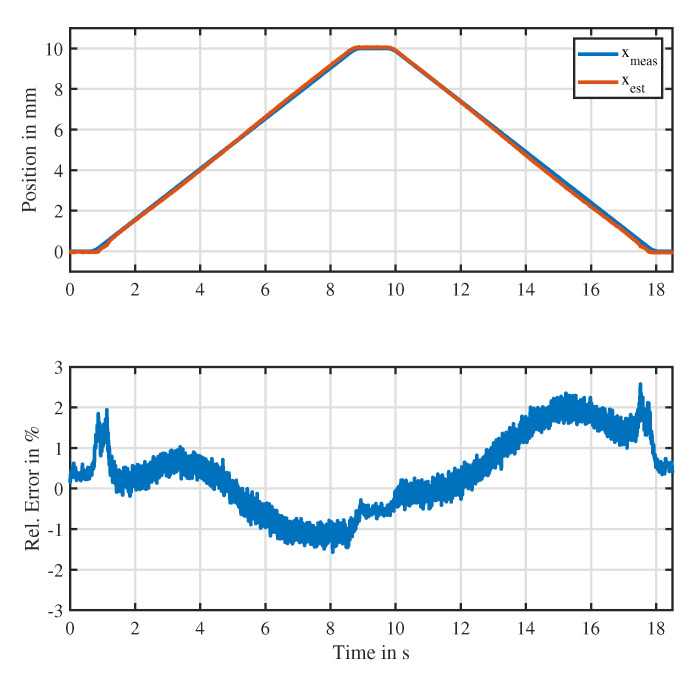
Estimation performance of the discussed approach when the actuator is driven with zero mean current. (**Top**): measured position compared to the estimated position; (**bottom**): relative error of the estimated position.

Figure 15 shows the comparison of the measured position to the estimated position in case the actuator is driven with zero mean current along with the relative error of the position estimate, which was related to the maximum stroke of 10 mm of the actuator. It is visible that the proposed position estimator based on a polynomial model was able to track the position with relative errors less than 2.7%.

**Figure 16:**
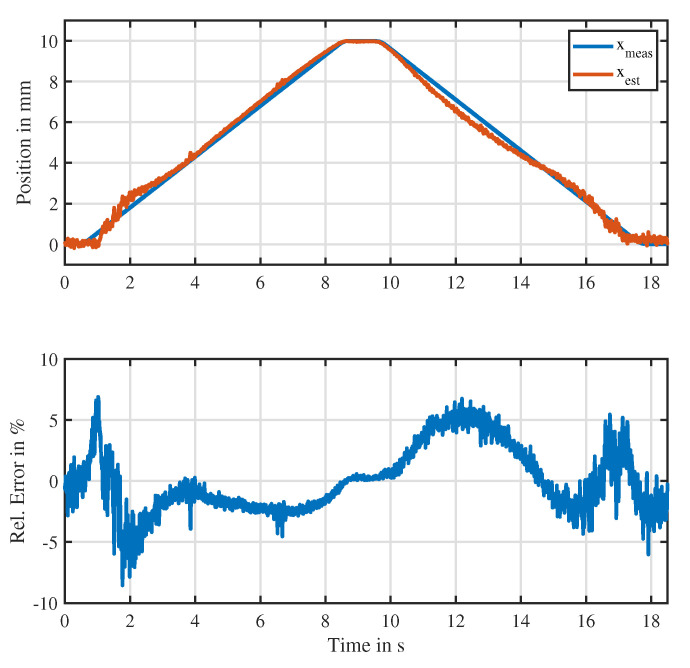
Estimation performance of the discussed approach when the actuator is driven with full mean current. (**Top**): measured position compared to the estimated position; (**bottom**): relative error of the estimated position.

Figure 16 illustrates the estimation performance in case the actuator is driven with nominal mean current. In this case, the proposed approach based on a MLP neural network was able to reconstruct the position with a relative error less than 8% without showing any ambiguities. Therefore, ambiguities present in the differential inductance could be resolved by the discussed approach. While the estimator showed relative errors less than 2% at the minimum and maximum stroke, intermediate positions showed higher relative errors with different kinds of causes. At positions next to 2.5 mm, present at the time intervals [1 s, 3 s] and [15 s, 17 s], the estimate showed a deviation and exhibited a low SNR. This is due to the fact that the differential inductance at this point exhibited an extremum, which led to a loss of sensitivity towards position variations. Therefore, the estimator could only rely on the information of the parallel resistance $R_{p}$, a parameter that showed large variance and low SNR. Moreover, on the forward direction of the position estimation, present at the time interval [4 s, 8 s], there existed a negative relative error of −3%. On the way backwards at the same positions, present during the time interval [10 s, 14 s], the estimator exhibited a positive error of 5%. Such direction-dependent errors can be explained by the magnetic hysteresis of both parameters $R_{p}$ and $L_{d}$ that was neglected during modeling. In fact, at the minimum and maximum stroke, the estimator exhibited little relative errors since at these end positions there was no significant influence of the hysteresis. Moreover, given the characteristic shown in Figure 13 at a nominal current of 400 mA, the sensitivity of the differential inductance at the highest position x=10 mm was very high.

## 5. Conclusions

In this work, the problem of ambiguities in position estimation for solenoid actuators based on the differential inductance has been addressed. In order to achieve a uniqueness of the position estimate, the eddy current losses inside the actuator are evaluated when a PWM voltage is applied. In particular, at the PWM switching instants, the current shows considerable cusps, which can be modeled by a lumped parameter model involving a resistor in parallel to the main inductor. This resistor exhibits a monotonous dependency on the position, thus allowing a unique position estimate.

The discussed approaches for the identification of the differential inductance as well as the parallel resistance are computationally lightweight and require a reduced sampling effort. In particular, the Integrator-Based Direct Inductance Measurement technique, used for estimation of the differential inductance, requires seven samples per PWM period while the proposed parallel resistance estimator requires three samples per each PWM period. Thus, AD converters with low sampling rate and low performance microcontrollers can be used, thereby allowing the implementation of sensorless position estimation on solenoids used in cost-critical systems. In contrast to state-of-the-art works, the position is estimated by means of a small neural network, that weights and merges the different position-dependent parameter information. Therefore, the position estimator benefits from high accuracy and high SNR due to the identified value of the differential inductance and exhibits no ambiguities due to the identified value of the parallel resistor.

Experimental validation involving a use case scenario for sensorless end-position detection proves the uniqueness, accuracy as well as high SNR of the approach. Nevertheless, the estimate still shows an error that is related to the negligence of hysteresis in the obtained characteristics. Therefore, identifying and compensating the hysteresis is a promising research aspect for further increase of accuracy. Moreover, the effect of temperature dependency has been neglected during the experiments. In industrial applications, the actuators are usually exposed to environments with large temperature variation, influencing the magnetic permeability and electrical conductivity of the used materials. Thus, further investigations on the temperature dependency of the position estimation need to be conducted.

## 6. Patents

The IDIM method has been submitted for patenting and reference can be found in [25].

## Figures and Tables

**Table 1:** Parameters of the solenoid actuator ITS-LZ 1949 from Red Magnetics as well as implementation parameters of the IDIM technique and the parallel resistor estimator.

Parameter	Value
Nominal Voltage	24 V
Series Resistance	23 Ω
Nominal Stroke	10 mm
Max. Force	6 N
PWM Frequency	1000 Hz
Reset time $t_{r}$	50 μs
Measurement time $t_{\delta ,0}$	−12 μs
Measurement time $t_{\delta ,1}$	20 μs
Low pass filter frequency	20 Hz
